# Personality Disorders in Time of Pandemic

**DOI:** 10.1007/s11920-020-01204-w

**Published:** 2020-11-10

**Authors:** Emanuele Preti, Rossella Di Pierro, Erika Fanti, Fabio Madeddu, Raffaella Calati

**Affiliations:** 1 Department of Psychology, University of Milan-Bicocca, Piazza dell’Ateneo Nuovo, 1, 20126 Milan, Italy; 2 Department of Adult Psychiatry, Nîmes University Hospital, 4 Rue du Professeur Robert Debré, 30029 Nîmes, France

**Keywords:** Personality disorders, Mental health, Pandemic, Epidemic, COVID-19

## Abstract

**Purpose of Review:**

We report evidence on the negative psychological effects of pandemics in people with personality disorders (PDs) and on the role of personality pathology in compliance with mitigation-related behaviors. Considering the paucity of studies, after a description of the main features of PDs, on the basis of the current literature on pandemic and quarantine mental health impact, we trace some clinical hypotheses.

**Recent Findings:**

Paranoid traits and detachment (cluster A) might lead to worse psychological outcomes. Cluster B patients may show more intense stress-related reactions and react strongly to social distancing, especially considering borderline personality disorder. Cluster C patients might be particularly prone to anxiety and stress due to fear of contagion and may be less flexible in adaptation to new routines. Evidence on compliance with mitigation measures is mixed, with lower compliance in cluster B patients and higher in cluster C ones.

**Summary:**

We suggest that PD patients might be particularly affected by pandemics. Furthermore, they might react differently, according to their main diagnosis. Similarly, compliance with mitigation measures may differ according to specific PDs. Our results should be considered as a starting point to reflect on therapeutic strategies to be adopted in the post-COVID-19 situation.

## Introduction

Coronavirus disease 2019 (COVID-19) represents a major threat to public health. After the first outbreak in Wuhan (Hubei, China) in December 2019 and the declaration of a global pandemic by the World Health Organization (WHO) on March 11, 2020, the virus is still consistently spreading in 216 countries, with more than 29 million patients infected and more than 900,000 deaths by the time we are writing.^1^

In light of these events, psychological responses to pandemic situations have become a major topic of interest, both from a research and clinical point of view. Empirical studies and scientific reviews about the consequences of pandemics and quarantine measures in terms of mental health are concordant in concluding that both short- and long-term negative psychological effects can be observed in community people [1, 2] and healthcare workers [3]. Moreover, a recent review suggests relevant negative psychological effects of pandemics also in people with pre-existing mental health disorders [4••]. However, little is known on the specific effects of pandemics on patients with personality disorders (PDs).

Empirical literature on the effect of pandemic on patients with personality pathology, however, lacks. PDs are severe mental disorders that manifest with moderate to severe impairment in both self and interpersonal functioning [5–7]. That is, such patients show serious difficulties in emotion regulation and interpersonal relationships. Since pandemic showed to be a stressful event with consequences on emotions [8] and social life [9], we can expect that it might represent a relevant risk factor for the exacerbation of negative psychological consequences specifically connected to personality pathology. Furthermore, the pathological personality traits showed by individuals with PDs might pose difficulties in compliance with mitigation measures needed during pandemic outbreaks.

For these reasons, we performed a narrative review of studies investigating pandemic-related mental health issues and in particular including only issues related to PDs and personality traits. Considering the paucity of studies on this topic, after a description of the main features of PDs, on the basis of the current literature on pandemic and quarantine mental health impact, we aim at tracing some clinical hypotheses on the negative psychological effects of pandemic situations in people with PDs. Furthermore, we aim at investigating the role of personality pathology in compliance with mitigation-related behaviors.

## Methods

We searched for original studies published in PubMed until July 2020 using the medical subject headings (MeSH) “pandemic,” “oubreak,” “COVID*,” “lockdown,” “quarantine,” “SARS,” “influenza,” “flu,” “MERS,” “ebola” combined with “mitigation measures,” “compliance,” and “adherence” and with “personality,” “trait,” “temperament,” and “personality disorder.” We considered only studies published in the English language. We also reviewed the list of references to identify other studies of interest.

Considering the paucity of studies on this topic, we adopted the following steps in the description of results: (1) we provided a description of the main features of PDs for each cluster; (2) we mentioned the main literature investigating the association between PDs of each cluster and other psychiatric disorders; (3) on the basis of the literature on pandemic (e.g., [4••]) and quarantine (e.g., [1••]) mental health impact, we hypothesized a plausible relation between PDs of each cluster and specific psychological/psychiatric outcomes, as well as problems in compliance with mitigation measures.

## Results

We present a synthesis of our results in Table 1. The table reports a brief description of PDs, the traits and symptoms that are likely to play a role in response to pandemics, and the negative psychological outcomes due to pandemics and quarantine that we hypothesize might be bolstered by personality pathology.

**Table 1:** Personality disorder symptoms that can be risk factors for the development of pandemic-related negative psychological impact

PDs clusters	PDs	PDs symptoms	Alternative DSM-5 model for PDs	Pandemics and quarantine psychological impact
Cluster A The “odd, eccentric” cluster	Paranoid/schizoid/schizotypal	Suspiciousness or paranoid ideation (paranoid, schizotypal) Perception of threats (paranoid, schizotypal) Lack of interest/pleasure in relations (schizoid) or lack of close friends (schizotypal) or social anxiety (schizotypal)	Psychoticism Detachment	- Anxiety symptoms* - Depressive symptoms* - Avoidance behaviors such as minimizing direct contact (Q) - Delay in return to normality (Q) - Disruption of social networks (Q)
Cluster B The “dramatic, emotional, erratic” cluster	Borderline/narcissistic/histrionic/antisocial	Fear of abandonment (borderline) Relationship instability (borderline) Impulsivity (borderline, antisocial) Suicidality (borderline) Affective instability (borderline) Chronic emptiness (borderline) Intense anger (borderline) Paranoid ideation (borderline) Need to be the center of attention (narcissistic, histrionic) Irritability and aggressiveness (antisocial)	Negative affectivity Disinhibition Antagonism	- Anxiety symptoms* - Depressive symptoms* - Impulsivity* - Anger* - Suicidality (intense suicidal ideation and suicide) (P) - Extreme fear (P) - Emotion dysregulation (Q) - Emotional exhaustion (Q) - Alcohol abuse or dependency symptoms (Q) - Irritability (Q) - Numbness (Q) - Worsening of eating disorders symptomatology (P) - Emotional eating (P)
Cluster C The “anxious, fearful” cluster	Avoidant/dependent/obsessive-compulsive	Avoidance of interpersonal contacts and new activities (avoidant) Preoccupation in social situations (avoidant, dependent) Inhibition and lack of self-confidence (avoidant, dependent) Difficulties in making everyday decisions or doing things on his or her own (dependent) Fear of loss of support or approval (dependent) Devotion to work and productivity to the exclusion of leisure activities and friendships (obsessive-compulsive) Overconscientiousness, scrupulosity, and inflexibility about matters of morality (obsessive-compulsive)	Negative affectivity Detachment	- Post-traumatic stress symptoms* - Anxiety symptoms* - Depressive symptoms* - Insomnia* - Serious worries about physical health (P) - Compulsive symptoms (P) - Extreme fear (P) - Acute stress disorder (Q) - Avoidance behaviors such as minimizing direct contact (Q) - Delay in return to normality (Q) - Disruption of social networks (Q)

*Impact reported for both pandemics (e.g., [4]) and quarantine (e.g., [1]); *P*, pandemic impact; *Q*, quarantine impact

### Cluster A Personality Disorders

#### Negative Psychological Impact

Patients with cluster A PDs are usually highly introverted, emotionally detached, and hypersensitive to interpersonal threats due to paranoid tendencies (e.g., [10, 11]). As a consequence, patients with paranoid, schizoid, or schizotypal PDs may be at higher risk for serious psychological issues during pandemic emergencies.

In pandemic situations, other people represent potential threats to one’s own survival, and intense fear for contagion is a natural psychological reaction to these events [12]. In cluster A PDs, patients’, however, fear of contagion may intensify pre-existing paranoid tendencies and exacerbate suspect toward others, by supporting pre-existing persecutory representations of others. In fact, within Kernberg’s object relation theory framework, personality pathology is sustained by massive use of splitting defense mechanism which lead patients to divide the world in all-good and all-bad objects, producing unstable, polarized, dissociated, fragmented, and distorted views of both self and significant others [13, 14]. According to this view, projection into others of one’s own negative parts contributes to a persecutory view of others, typical of patients with paranoid tendencies [15].

Moreover, it is reasonable to expect that intensifications of paranoid tendencies may lead cluster A PDs patients to experience high levels of psychological distress, depressive feelings, and anxiety symptoms. There is now evidence that the COVID-19 pandemic caused a sharp increase in the prevalence of anxiety and depressive problems in both the general population [16] and clinical samples [17]. Furthermore, 20.9% of outpatients with pre-existing psychiatric disorders reported a deterioration in their condition during the COVID-19 pandemic [17]. As for cluster A PDs, high levels of depressive feelings in patients may be linked to presumed perception of untrustworthy others [18], and their constant need of keeping a watchful eye on others may be responsible for high psychological distress and anxiety (e.g., [19]).

Furthermore, claims that the coronavirus pandemic would have originated in laboratory have emerged during the COVID-19 outspread [20–22]. Since paranoid PD patients show a pervasive and long-standing suspiciousness and mistrust of others, they may be particularly suitable for believing and spreading conspiracy theories about pandemics. This hypothesis is in line with recent findings [23] showing a positive association between paranoid traits and conspiracist ideation. Moreover, the wide use of “war metaphors” by healthcare workers and journalists during the COVID-19 pandemic may have a detrimental effect in these patients, by fostering splitting defense mechanisms and by strengthening interpretation of external reality in terms of winners and losers [24].

Finally, social distancing measures may also have peculiar psychological impacts on cluster A PDs patients. In a sense, these recommendations may bolster their proneness to introversion, social withdrawal, and isolation (e.g., [25, 26]). For instance, it is plausible to expect that patients with paranoid, schizoid, or schizotypal PDs would significantly reduce their social contacts during pandemic emergencies, with great difficulties in restoring them when lockdown measures end. As a consequence of their prevailing traits of detachment [5], cluster A PDs patients are likely to show reduced emotional well-being [27••] and higher levels of depression, anxiety, and stress [28••] during pandemics. Furthermore, social isolation, and limitations in contacts with mental health professionals, may lead patients to experience feelings of depression and loneliness, especially in the case of schizotypal PD. Albeit socially isolated, indeed, schizotypal patients usually desire to have social contacts [29, 30].

#### Compliance With Mitigation Measures

We may expect that some dispositional tendencies, such as paranoid thoughts and social withdrawal, make cluster A PDs patients—paranoid PD patients particularly—prone to high compliance with mitigation measures. In line with this hypothesis, some studies [31, 32] have recently shown that people high in extroversion have difficulties in keeping social distancing and in adhering to other mitigation measures. As a result, we may expect that the higher the detachment (which is opposed to extroversion), the higher is the tendency to be compliant with social distancing and mitigation measures during the outbreak. However, these results were not replicated by another study describing a positive correlation between extroversion and preparatory behaviors (i.e., face mask, hand sanitizer, toilet paper, food, travel cancelation) [33].

On the other hand, schizoid individuals suffer from communication and cognition impairments and have an unconventional life-style [34, 35], which often leads them to live as marginalized subjects. Similarly, schizotypal individuals have an impaired cognition, being deficient in attention, executive function, abstraction and memory, suffering from deficits in verbal learning, and lacking cognitive flexibility [36, 37]. Moreover, they have an impaired self-monitoring function, leading them to struggle to differentiate inner thoughts and reactions compared to those generated by the external [38], and often suffer from auditory hallucinations, delusions, and magical thinking [39, 40]. These lack of functioning in several areas, paired with the high prevalence of cluster A PDs disorders in homeless people [41–44], might prevent them to be compliant to mitigation measures and especially to hygiene norms.

### Cluster B Personality Disorders

#### Negative Psychological Impact

Cluster B PDs have unstable interpersonal relationships, and show behaviors that are overly emotional, impulsive, dramatic, and erratic. Since their vulnerabilities might be attributable to underlying hyper-responsiveness to stress and hypersensitivity to threat (e.g., [45, 46]), we expect that pandemic emergencies would seriously impact mental health in these patients. In line with our expectation, negative affectivity was found to be a risk factor for reduced emotional well-being during the COVID-19 pandemic [27••]. Moreover, negative affectivity was associated with high levels of depression, anxiety, and stress [28••]. Coherently, neuroticism, which is the adaptive corresponding trait of negative affectivity, was linked to reduced psychological well-being [47], more concerns, and longer pandemic duration estimates [33].

Pandemic emergencies force people to drastically reduce contacts with significant others for a quite long time, with relevant consequences in terms of disruption of daily life routines and conditions of social isolation. Being forced to keep distance from significant others (e.g., parents, partners, friends) may be particularly critical for both borderline and histrionic patients. In fact, borderline and histrionic PDs share a strong need for emotional and physical proximity with others [48, 49]. Moreover, borderline patients suffer from abandonment fears, rejection sensitivity, and paranoid preoccupations under conditions of stress [50, 51]. In this sense, the experience of lockdown may be particularly exhausting for these patients. It is reasonable to expect that such isolation may trigger negative feelings about oneself and the others, with an intensification of interpersonal conflicts, due to misinterpretation of others’ distance in terms of abandonment or disinterest. As a consequence, borderline patients perceiving distance of others in terms of abandonment might be more likely to engage in substance misuse as a form of self-medication [52–54] and in both suicidal behaviors and non-suicidal self-injury to cope with loneliness [55]. In addition, emotion dysregulation and difficulties in reading others’ emotional expressions (e.g., [56]) might lead borderline patients to read in advance subtle emotional expressions of fear or anxiety in their significant others and this, in turn, might trigger intense reactions such as anger outbursts, high irritability, and impulsive behaviors (e.g., maladaptive eating behaviors). This expectation is in line with a recent study [57] showing that cyclothymic temperament, which is best expressed by cluster B patients [58], was related to greater psychological distress during the coronavirus outbreak. Researchers have found evidence that conscientiousness was related to higher psychological well-being [47], less pandemic duration estimates, less concerns in general, and more concerns about community [33]. Since conscientiousness is opposed to traits of disinhibition which, according to the Alternative Model of Personality Disorders (AMPD; [5]), are distinctive of borderline patients, we may hypothesize that such patients may experience less psychological well-being during the outbreak, estimate longer pandemic duration, and do not care about community. As for histrionic PD patients, they usually show attention-seeking behaviors and an excessive need for attention [5]. Again, the mass indoor quarantine may lead histrionic patients to feel deeply alone, with consequent high levels of anxiety and depressive feelings [59].

Narcissistic features, on the contrary, may prevent patients from experiencing maladaptive psychological outcomes during pandemic outspreads. In particular, grandiose narcissistic individuals are self-absorbed (e.g., [60, 61]), socially cold and dominant (e.g., [62]), and base their self-view on agentic traits rather than on communal ones (e.g., [63]). In a sense, such attributes might protect narcissists from experiencing psychological distress during pandemics. Gupta and Parimal [47], however, have recently found that traits of agreeableness relate to greater psychological well-being during pandemics. Since both narcissistic and antisocial patients, according to the AMPD [5], share high traits of antagonism (which is opposed to agreeableness), they might show poor psychological well-being during pandemics. Furthermore, recent studies show that pathological narcissism include both grandiose and vulnerable manifestations [64], and that psychological distress usually relates to vulnerable traits [62]. Therefore, we might expect that forced social isolation limits narcissists’ occasions to search for admiration from others, with consequent feelings of hopelessness which are typical of vulnerable manifestations of narcissism. Finally, social isolation might lead antisocial patients to experience psychological distress by limiting their chance to express their hostility toward others, as they usually do.

#### Compliance With Mitigation Measures

Overall, we may expect poor compliance with social distancing and mitigation measures in cluster B PDs patients. For instance, impulsiveness may affect seriously the ability of borderline patients to keep social distancing. Indeed, conscientiousness, as opposed to disinhibition, was positively linked to social distancing, hand washing, and hygiene [32, 65]. In line with these findings, Brouard and colleagues [31] noted a positive link between conscientiousness and adherence to mitigation measures, while Aschwanden et al. [33] reported the trait to be associated with more precautions. So, people with higher disinhibition may be prone to act less social distancing, hygiene, and to be less adherent to mitigation measures.

We might expect that both narcissistic and antisocial patients are poorly motivated to follow mitigation measures. In fact, narcissistic patients have a grandiose self-view [66] and this, in turn, may lead them to think they are exempt from mitigation norms. Moreover, antisocial PD comprises failure to conform to social norms [5] and disregard for others [67]. These expectations are also supported by Aschwanden et al. [33] showing that agreeableness (which is opposed to antagonism) is positively related to social distancing during the outbreak.

### Cluster C Personality Disorders

#### Negative Psychological Impact

Patients with cluster C PDs display anxious and fearful thinking and behaviors. These features may make patients with avoidant, dependent, or obsessive-compulsive PDs at high risk to develop serious psychological outcomes during pandemic outspreads. In particular, these patients may be particularly sensitive to anxious feelings originating from fear of contagion during pandemics. In fact, during the COVID-19 pandemic, anxiety and depression were found to be positively associated with fear of infection about oneself and loved ones [16]. Since traits of anxiety are central in avoidant PD according to the AMPD [5], and patients with avoidant PD “feel fearful, apprehensive, or threatened by uncertainty” ([5]; p. 766), these patients may suffer from serious anxiety symptoms during pandemics. In fact, pandemic emergencies force us to live in a constant state of uncertainty (e.g., job uncertainty).

Avoidant PD, along with the other cluster C PDs, is characterized also by traits of detachment, and recent findings suggest that such traits relate to reduced emotional well-being [27••], depression, anxiety, and stress [28••] during pandemic outbreaks. Moreover, Bacon and Corr [68] found that behavioral inhibition was related to higher depression and anxiety in response to the COVID-19 pandemic. Some studies on the effect of adaptive personality traits on psychological reactions to pandemics, however, do not confirm such associations. In fact, extroversion (which is opposed to detachment) was found to be linked to less psychological well-being [47] and greater concerns [33] during pandemics.

As well known, dependent PD patients are unable to be alone and they rely on others for reassurance and support [5]. Therefore, they may experience high levels of anxiety, psychological distress, and sleep disturbances in response to intense worries about others’ survival: The idea that the people they depend on can get sick and no longer be available would be intolerable for them. In this sense, dependent patients may be at high risk to develop depressive symptoms and post-traumatic conditions if their significant others are affected by the disease, as well as to show post-traumatic conditions in case of serious medical conditions (e.g., conditions requesting hospitalization) or death of significant others. As discussed above, studies have demonstrated that negative affectivity is a risk factor for reduced emotional well-being [27••], depression, anxiety, and stress [28••], and neuroticism was related to less psychological well-being [47], more concerns, and longer duration estimates related to COVID-19 [33]. Besides anxiety and depression, evidence of post-traumatic stress disorder in the post-illness stage of previous coronavirus epidemics was reported [69].

Patients with obsessive-compulsive PD may be also particularly sensitive to anxiety and depressive symptoms during the coronavirus emergency, since they are inflexible and show excessive need for control, extreme perfectionism, and excessive devotion to work. Such dispositions may lead these patients to experience high levels of both anxiety and depressive symptoms in response to the need of facing disruptions of daily life routines (e.g., [70]). In fact, the lockdown period forced individuals to adopt highly flexible working models, and to full-time cohabitation for weeks. Moreover, obsessive-compulsive PD individuals show difficulties in coping with uncertainty [71], and are intolerant to changes and deviations in their routine (e.g., [72]). In line with our assumptions, anxious and depressive temperament, which are best expressed by cluster C patients [58], are related to greater psychological distress during coronavirus outbreak [57].

#### Compliance With Mitigation Measures

Our expectations regarding compliance with mitigation measures in cluster C PDs patients are somewhat mixed. On the one hand, we may expect high compliance with mitigation measures, since dispositions toward high anxiety and fearful thinking and behaviors of these patients well fit in with the governments’ guidelines for pandemic mitigation strategies. On the other hand, however, these patients may show difficulties in following some mitigation measures due to their rigidity and inflexibility, as in the case of obsessive-compulsive PD patients [73, 74].

Our hypotheses are not univocal also when considering the role of pathological personality traits describing cluster C PDs, according to the AMPD [5]. For instance, studies on the association between detachment and adherence to mitigation measures are not available, and empirical findings on the role of extroversion (which is opposed to detachment) are mixed. Extroversion has been found to be negatively associated with social distancing [32] and adherence to other mitigation measures [31]. On the contrary, Aschwanden et al. [33] found a positive association between extroversion and preparatory behaviors. The same is for negative affectivity. In fact, Abdelrahman [65] found that neuroticism is related to greater social distancing, whereas Brouard et al. [31] showed that it was negatively correlated to COVID-19 mitigation behaviors. Consistent with Brouard et al. [31], however, Aschwanden et al. [33] found that emotional instability related to fewer precautions. After all, previous studies showed that people who are emotionally unstable appraise their coping ability not enough to face acute stress, and this may lead them to freeze/not act [75].

## Conclusion

With this review, we aimed at investigating the negative psychological impact of pandemic situations in patients with personality pathology. Since impairments and manifestations of personality pathology differ according to the specific type of disorder, we hypothesized that pandemics might affect differently patients, according to their main PD diagnosis (Table 1).

We based our hypotheses on clinical understanding of PDs, and we supported our statements by mentioning some recent empirical findings. In fact, at the moment, empirical studies on the effects of pandemics on patients with PD diagnosis are lacking. There are, however, some available studies inspecting the effect of pathological personality traits [27, 28], adaptive personality traits (e.g., [32]), and temperamental dimensions (e.g., [57]) on dysfunctional reactions to pandemics.

Our review suggests that PD patients might be particularly affected by pandemic situations. Furthermore, they might react differently to pandemics, according to their main diagnosis and related manifestations. Considering cluster A PDs, we hypothesize that paranoid traits may foster conspiracy theories and negative views of the other and that detachment might interact with quarantine measures in worsening social isolation. Cluster B patients may show stress-related reactions, including impulsive and risky behaviors. This is particularly true for borderline patients: difficulties in emotion regulation and fear of abandonment and rejection might render social distancing particularly painful for these patients. On the other hand, we might expect that narcissistic patients, due to their self-absorption, might be relatively protected from the negative effects of isolation, although difficulties in nourishing their grandiose view of self. Finally, cluster C patients might be particularly prone to anxiety and stress reactions in response to fear of contagion and may show serious disturbances in response to intense worries about others’ survival. Furthermore, rigidity and intolerance to change might interfere with the need of flexible adaptation to new routines.

Similarly, we showed that compliance with mitigation measures and social distances may differ significantly according to specific PDs. Detachment might be a factor that makes cluster A patients more prone to following mitigation measures. However, severe cognitive and functional impairments in these patients pose a threat to following organized behaviors. Impulsiveness and disinhibition of cluster B patients might render them less compliant with mitigation measures, and this might be particularly true for patients with narcissistic and antisocial personality disorder, due to their grandiose self-view and disregard of others. Finally, cluster C patients might present mixed levels of compliance. On the one hand, due to anxiety and fear of contagion, they might be more probe to follow prescribed measures; however, rigidity (especially in obsessive-compulsive PD) might pose difficulties in changing behavioral routines and adapting to the mitigation behaviors.

As a final note, limitations in the provision and accessibility of mental health services can have a particular impact on patients with PDs. Mitigation measures and the global emergency led to partial or total disruptions of some forms of treatment [4••]. Some of these treatment options have a particular relevance for complex mental health conditions such as PDs. Reduced inpatient treatment capacities and early discharges [76–79] might have detrimental effects on patients with PDs. Also, group interventions (e.g., skills training groups or peer support meetings) are a treatment component in many therapies for patients with PDs, and their reduction or cancelation [80, 81] can affect the course of treatment for these patients.

To the best of our knowledge, this is the first review attempting to trace some hypotheses on PD patients’ reactions during epidemics. The paucity of related studies represents a limitation of this review. In particular, not only negative impact but also symptomatologic amelioration may have been registered, especially during the initial phase of the lockdown, revealing adaptive coping strategies in these patients [82]. The present results should thus be considered as a starting point to reflect on therapeutic strategies to be adopted in the post-COVID-19 telepsychology and telepsychiatry revolution [83, 84].

## Footnotes

1 Data are provided by the World Health Organization (https://www.who.int/emergencies/diseases/novel-coronavirus-2019).
